# Supplementary material for: The Nephroprotective Effect of Nitric Oxide during Extracorporeal Circulation: An Experimental Study
Source: Biomedicines. 2024 Jun 12;12(6):1298. doi: 10.3390/biomedicines12061298 (PMC11201384; doi:10.3390/biomedicines12061298)
Supplement: Supplementary file 1 [file biomedicines-12-01298-s001.zip › biomedicines-3001934-supplementary.pdf]

The Nephroprotective Effect of Nitric Oxide during Extracorporeal Circulation: An Experimental Study

Online supplementary data

Study design, randomization, and blinding:

This study's methodology was developed in accordance with international guidelines for randomized controlled trials. The sequence of events and study methodology are presented below.

1. The recruitment of animals for the study.
2. Animal examination by a veterinarian and admission to participate in the study.
3. Randomization.
4. The induction of anesthesia and the onset of mechanical ventilation.
5. NO (Nitric Oxide) conditioning or the standard protocol without NO treatment during the entire main phase of the experiment.
6. Anesthesia according to a standardized protocol.
7. Conducting cardiopulmonary bypass (CPB) or CPB + circulatory arrest (CA) according to the protocol standardized and accepted in clinical practice.
8. Tracking clinical events and characteristics; obtaining serial perioperative measurements of biochemical variables.
9. The collection of biopsy samples.
10. The conservation of biopsy samples.
11. The withdrawal of animals from the experiment.
12. Morphological and histochemical evaluations of clinical events.

Randomization was carried out using sealed opaque envelopes. The envelopes were prepared prior to the recruitment of animals for the study, and their number corresponded to the

estimated sample size. The processing distribution was prepared by an independent operator (a researcher not involved in the study) and hidden in opaque sealed envelopes that were consecutively numbered. Each envelope contained one code name: “CPB”, “CPB+NO”, “CPB+CA”, or “CPB+CA+NO”. On the morning of the experiment, when an envelope was opened, its contents were not disclosed. Thus, the animals were assigned to the group of perioperative NO conditioning with CPB or CPB+CA or to the group that received the standard-protocol CPB or CPB+CA in a 1:1:1:1 ratio. All animals were randomized into four equal groups of six sheep:

1. CPB group: the standard protocol of mechanical ventilation and cardiopulmonary bypass (CPB) adopted in clinic was carried out;

2. CPB+ NO group: NO was delivered immediately after tracheal intubation through the circuit of the ventilator at a dose of 80 ppm, and then at the start of CPB, NO was delivered to the extracorporeal circulation circuit at a dose of 80 ppm throughout the entire period of CPB (90 min); after weaning the subject from CPB, the NO supply continued through the circuit of the ventilator at a dose of 80 ppm for 1 hour;

3. CPB+CA group: the standard protocol adopted in clinic for mechanical ventilation, CPB, and hypothermic circulatory arrest was carried out;

CPB+CA+NO group: NO was delivered immediately after tracheal intubation through the ventilator circuit at a dose of 80 ppm; then, at the start of CPB, NO was delivered to the extracorporeal circulation circuit at a dose of 80 ppm until hypothermic circulatory arrest was initiated. However, during non-perfusion hypothermic circulatory arrest (when the target body temperature—an esophageal temperature of 30°C—was reached, the perfusion index decreased to 1 L/min/m2, and the descending aorta was occluded for 15 min), NO delivery was not performed. Upon completing CA, NO delivery to the extracorporeal circuit at a dose of 80 ppm was resumed and maintained until the end of warming and when the target temperature was

reached (the cumulative duration of CPB and the period of circulatory arrest was 90 min). After weaning the subject from CPB, NO was supplied through the ventilator circuit at a dose of 80 ppm for 1 hour.

The specialists involved in conducting the experimental studies, obtaining and interpreting the results, did not know about the nature of the intervention until the completion of the study. The investigator responsible for NO delivery and monitoring remained unblinded and was responsible for masking gas delivery and monitoring systems and for maintaining the randomization codes.

Methods of anesthesia and cardiopulmonary bypass:

The experiment began with the sevoflurane mask induction of anesthesia. After reaching the target level of anesthesia, preoperative preparation comprising shaving and processing the surgical area was performed. To do this, the animal was placed in a lateral position and fixed with soft ties. To achieve surgical access, hair was removed from the right anterolateral surface of the animal's body; to provide peripheral and central venous access, as well as arterial access, hair was removed from the front surfaces of the neck and hind limbs. To carry out initial infusion therapy and induce anesthesia using an aseptic technique, catheterization of the great saphenous vein of the hind limb was performed with an 18 G catheter. Infusion therapy was initiated with Sterofundin solution (B. Braun, Germany). Atropine sulfate 0.5 mg and chloropyramine 20 mg were administered intravenously. Before the induction of anesthesia, pulse oximetry and electrocardiogram (ECG) monitoring were provided, with ECG electrodes placed on incisions in the skin of the anterolateral surface of the body. When monitoring the main vital functions and after premedication, general anesthesia was induced by the fractional administration of propofol 1% at a dose of 5 mg/kg; while maintaining spontaneous breathing, direct laryngoscopy followed by orotracheal intubation was performed. To do this, the animal was laid down and fixed in the lateral position, and its head was left free. One tie was positioned

on the upper incisors so that the head lay flat and the neck of the animal was unbent. By palpating the skin along the midline of the neck from the middle of the thyroid cartilage, moving 3–4 cm caudally, the trachea was found and fixed. Tracheal intubation was performed with an endotracheal tube measuring 6.5 mm and an introducer. After confirming the position of the endotracheal tube, the introducer was removed, and the animal was connected to a mechanical ventilator. Mechanical ventilation was performed with a Puritan Bennett 760 ventilator (USA) using a modified breathing circuit with a tidal volume of 8 mL/kg and a respiratory rate of 20 breaths per minute in Continuous Mandatory Ventilation (CMV) mode with a controlled volume, a fraction of inspired oxygen (FiO2) of 50% and a positive end-expiratory pressure of 5 cm H_2O. In the interventional groups comprising experimental animals that were conditioned with nitric oxide, immediately after tracheal intubation, the delivery of nitric oxide was initiated through a modified breathing circuit that allowed for NO supply and sampling of the gas–air mixture to analyze the fractional concentration of NO and NO_2. TIANOX, a device for plasma–chemical synthesis, was used for the synthesis, delivery, and monitoring of nitric oxide. Maintenance of anesthesia throughout the experiment was provided via the infusion of propofol at a dose of 5 mg/kg/h and fentanyl at a dose of 3.0 μg/kg/h. Neuromuscular blockade was achieved with pipecuronium bromide at a dose of 0.1 mg/kg. Throughout the experiment, extensive monitoring during anesthesia was used, including ECG monitoring, invasive blood pressure monitoring, pulse oximetry, continuous monitoring of end-tidal carbon dioxide ($etCO_2$), and thermometry using a Nihon Kohden BSM-4104A monitor (Japan); urine flow rate was also taken into account. The temperature sensor was placed in the esophagus. For invasive blood pressure monitoring and sampling for laboratory arterial blood gas tests, the common carotid artery was surgically harvested and catheterized with a 7F catheter. For infusion and inopressor therapy, the internal jugular vein was surgically harvested and catheterized with a 7F catheter. Blood gases were measured with a STAT PROFILE Critical Care Xpress analyzer

(Nova Biomedical, USA). Additionally, using this gas analyzer, the level of methemoglobin was monitored by reflectance photometry. Surgical access was achieved with right 4th–5th intercostal space thoracotomy. Cardiopulmonary bypass was performed using a Maquet Jostra HL20 CPB machine and a Kids D100 neonatal oxygenator (Dideco, Italy). The ram's total body area was considered equal to 1.1 m^2. CPB was carried out in a non-pulsatile mode. The CPB machine was connected according to the bicaval technique with the scheme "aorta–superior vena cava–inferior vena cava". The perfusion index was set at 2 L/min/m^2. Norepinephrine was used as a vasoactive drug in the animal groups at a dose of 0.05-0.1 μg/kg/h. The mean arterial pressure during CPB was maintained at 70 mm Hg. In groups of animals that did not simulate circulatory arrest, CPB was performed under normothermic conditions: the esophageal temperature was maintained at a level of 36–36.6°C. In the groups of animals in which circulatory arrest was simulated, CPB was carried out with hypothermia. After reaching the target esophageal temperature of 30°C, the descending aorta was occluded; thus, non-perfusion circulatory arrest was simulated for 15 minutes. The perfusion index was then reduced to 1 L/min/m^2. Next, the descending aorta was unclamped, followed by reperfusion and warming to 36.6°C. During the rewarming stage, the perfusion index was set to 2 L/min/m2. The cumulative duration of CPB in all groups (including the group which underwent CA during CPB, in which the duration of CA was also included) was 90 min. To ensure hypocoagulability during CPB, heparin at a dose of 3 mg/kg was used, maintaining an activated clotting time > 450 sec.

Nitric oxide conditioning:

The rationale for the dosing and duration of nitric oxide administration is provided below.

When choosing a dose and time of NO exposure, clinicians are to be guided by two basic principles:

1. The applied dose of nitric oxide and the time of its exposure should be safe for patients;

2. The applied dose of nitric oxide and the time of its exposure should be sufficient to provide potential protective effects.

Recommendations for dosing and perioperative nitric oxide therapy in cardiac surgery are currently developed only for cases of hypoxemic respiratory failure in newborns and young children during the surgical correction of congenital malformation of the circulatory system. Recommended doses range from 3–5 ppm to 40 ppm for reducing pulmonary vascular resistance to 50–80 ppm for providing hemodynamic support to the injured right ventricle [E1]. In the adult population, 20 ppm was an effective dose in various clinical scenarios such as acute respiratory distress syndrome and in heart transplant patients [E2].

The mechanisms of increasing NO bioavailability upon its exogenous administration are protein S-nitrosylation and an increase in the serum concentration of NO-NOx metabolites (nitrates, nitrites, S-nitrosothiol, N-nitrosamine, etc.), which serve as reserve donors of NO in the body [E3;E4]. The accumulation of these metabolites in organs subjected to ischemia–reperfusion explains the organ-protective effects of exogenous NO [E5]. In this regard, it seems reasonable to start NO therapy before the start of CPB. Starting NO delivery immediately after patient intubation and quite a long exposure to NO during the preparatory stages of surgical intervention (providing surgical access, harvesting the internal thoracic artery, and providing a cannulation scheme for CPB) will allow the target concentrations of NO and its metabolites to be reached not only in blood plasma but also directly in the visceral organs before the start of CPB and following ischemia–reperfusion injury. It is important to extend the exposure of NO therapy in the postoperative period to the period of early reperfusion after CPB, when the main part of organ damage occurs. Moreover, the persistence of plasma-free hemoglobin (fHb) after cardiac surgery was found in 83% of patients within 2 days after the intervention, and the peak

concentration of fHb associated with the development of AKI was observed 2 hours after weaning from CPB [E6]. At the same time, in some patients after cardiac surgery, the NO level and the expression of damage markers in kidneys and intestines returned to the initial level 6 hours after CPB [E7].

The safety of NO delivery at moderate doses was confirmed in patients with sickle cell anemia, and the continuous inhalation of NO at a dose of 40 ppm for 3.2 days in patients with multiple-organ damage did not cause side effects and improved the clinical state of patients [E8]. In our previous study, we tested a new concept of nitric oxide delivery with a fundamentally new technique (via the CPB circuit) and different endpoints (a decrease in CSA-AKI), suggesting extrapulmonary effects of nitric oxide [E9]. We investigated a NO dose of 40 ppm, drawing on data from previous studies showing that the inhalation of 40 ppm NO increased renal blood flow, the glomerular filtration rate, and urine output in an experimental model of phenylephrine-induced hypertension in pigs [E10]. However, we found that the concentration of NO metabolites (nitrates, nitrites, and the total concentration of metabolites—NOx) during the postoperative period was lower than baseline [E9]. Data from other researchers confirm that NO deficiency can develop during surgery and persist in the postoperative period, including through allogeneic transfusions [E11]. This was the reason for us to revise the dose of nitric oxide so that it would be increased and to continue NO therapy in the early postoperative period. In a study by Lei et al., NO therapy at 80 ppm was demonstrated to be effective and safe during CPB and up to 24 hours postoperatively in preventing postoperative acute kidney injury (AKI) and long-term chronic kidney disease (CKD) [E12]. Therefore, for this study, we chose a dose of NO equal to 80 ppm as optimal for the maximum implementation of organ-protective effects. When deciding on the time of exposure, it was extremely important to provide a therapeutic concentration of NO and its metabolites in organs and tissues before initiating ischemia–reperfusion injury. The start of NO inhalation at the start of CPB allows the

target concentration of NO donors to be achieved in the blood plasma; however, its concentration in target organs suffering from ischemia–reperfusion injury does not reach a level that activates organ protection pathways [E5]. Considering different organ-specific distribution rates of NO metabolites in blood/tissues, it may take 20 to 60 minutes of NO inhalation to reach a steady-state level. Moreover, the half-life of NO differs in different organs and blood [E5]. The start of NO delivery immediately after intubation makes it possible to increase exposure to therapy due to the time taken during the preparatory stage of surgical intervention (providing surgical access; harvesting grafts necessary for coronary artery bypass grafting; and providing a cannulation scheme for CPB), which averages from 40 to 60 minutes depending on the experience and qualifications of the operating surgeon. Thus, it is possible to achieve the target concentration of NO and its metabolites in organs and tissues even before the start of CPB and the formation of ischemia–reperfusion cycles.

Technique and equipment for NO conditioning:

The Research and Development Center for Physics of the Federal State Unitary Enterprise "Russian Federal Nuclear Center - All-Russian Research Institute of Experimental Physics" has developed and mass-produces a device for nitric oxide therapy AIT-NO-01 (the trade name is TIANOX). The device is registered in the Russian Federation as a medical device (registration certificate RU No. RZN 2020/10977, dated June 22, 2020). An International Certificate of Compliance with ISO 13485:2016, Medical Devices—Quality Management Systems, No. GKRU-0072-MD, for its production and for the product's life cycle has been received.

The device is designed to produce and deliver nitric oxide to the patient's breathing circuit and monitor the concentration of nitric oxide in the respiratory mixture supplied to the patient. Unlike existing devices for inhalation therapy with nitric oxide, TIANOX produces nitric oxide from gas discharge in the air. Other devices use nitric oxide in balloons, which are

filled at stationary chemical synthesis stations and delivered to the place of therapy. The traditional approach has a number of significant drawbacks, the most significant of which are complex logistics, the need to periodically purchase nitric oxide balloons, the limited shelf-life of gas in the balloons, and the high cost of therapy. TIANOX synthesizes nitric oxide from the air directly at the patient's bedside.

The practical implementation of gas discharge with parameters that satisfy medical application is a complex scientific problem. The prerequisite for this is the absence of ozone in the synthesized gas mixture and the absence of heating of the mixture, ensuring a high operating accuracy and the stable maintenance of the NO concentration; additionally, the electrode system should not change its characteristics over time, which would affect the formation of the discharge.

The TIANOX device solves this problem by generating a repetitively pulsed diffuse discharge excited in an atmospheric pressure air flow in a gap with a sharply inhomogeneous electric field. This discharge ensures the efficient synthesis of nitric oxide in a non-equilibrium, low-temperature plasma.

The special feature of the process is the fact that the electrode system in the plasma–chemical reactor is an element of an oscillatory circuit in which energy is released. This inclusion of the electrode system has a number of advantages, namely the formation of a discharge current profile and amplitude, the time limitation of energy release, and possible flexible control of the output characteristics of the plasma–chemical reactor in terms of nitric oxide generation. Voltage pulses of alternating polarity with highly stable electrical and time parameters are applied to the electrode system of the discharge chamber. This determines the accuracy and stability of the energy input in every pulse and, as a consequence, the stability of the NO concentration in the output mixture. The Nitric oxide concentration is regulated by changing the pulse repetition rate.

All units and blocks of the device are placed on a mobile trolley with a five-beam base. In the central part of the trolley, there is a retractable unit for stopping movement. A general view of the device is shown in Figure S1.

The NO-containing gas mixture synthesized in the generator enters the purification unit where, as a result of chemical adsorption, NO_2 is removed. Next, the mixture enters the patient's therapeutic circuit and mixes with the main respiratory flow, which is supplied from an external stimulator (a ventilator, a compressor, and an oxygen concentrator) or from the device's air supply unit. Immediately before delivery to the patient, a gas sample is taken from the breathing circuit for analysis in the monitoring unit. The operating principle of the monitoring unit is based on the use of electrochemical measuring sensors. The sensors are mounted on a sensor chamber in which a gas sample is supplied by a piston pump. The signals coming from the sensors are delivered to the conditioning amplifiers; next, they are converted into a digital form using an analog-to-digital converter and are sent to the microprocessor controller for processing. The microprocessor converts the signal into a measured value, taking into account the units of measurement (ppm), and displays the measurement results for NO and NO_2 on the display. To maintain measurement accuracy, the mode in which electrochemical sensors are purged with clean air is periodically switched on. The purge turns on automatically and does not require operator intervention. The purge interval is determined by the internal diagnostic system. After monitoring, the gas mixture is purified of nitrous gases in the neutralizer. Purification is carried out using the processes of NO_2 adsorption and the catalytic decomposition of NO.

Safety assessment of perioperative nitric oxide delivery:

The dosing and monitoring of NO were carried out using samples for the plasma–chemical synthesis of nitric oxide (Federal State Unitary Enterprise "Russian Federal Nuclear Center - All-Russian Research Institute of Experimental Physics", the Russian Federation) up

to a target concentration of 80 ppm. With this method of NO delivery, a significant amount of attention was paid to safety issues. Therapeutic NO concentrations are in the range of less than 100 ppm, and very short exposures to levels as low as 200 ppm can be fatal. NO is highly reactive; in the presence of oxygen (O_2), it undergoes a chemical reaction with the formation of nitrogen dioxide:NO_2: $2NO + O_2 = 2NO_2$. NO_2 is a highly toxic gas with a maximum allowed level of 5 ppm. The level of NO_2 in the gas–air mixture delivered to the circuit of the ventilator and the oxygenator was monitored continuously throughout the experiment. The rate of NO_2 formation depends on the concentrations of NO and O_2. This fact has important implications for NO delivery; a high NO concentration should be avoided, and NO and inspiratory O_2 should be used at the lowest clinically acceptable doses. The maximum allowed FiO_2 when using NO was calculated using the following formula: FiO_2 = (1 - required NO concentration / NO concentration in the source) x 100%. Thus, in the study, the maximum allowed FiO_2 for use in the O_2/air mixer of the CPB machine could be as high as 100%.

NO is one of several substances usually listed as methemoglobin formers; therefore, to exclude poisoning during the study, the methemoglobin (MetHb) level in the blood was monitored. The methemoglobin level was monitored in peripheral blood by reflectance photometry, using a Stat Profile CCX gas analyzer (Nova Biomedical, USA). Collecting blood samples for biochemical studies was carried out throughout the entire period of the research discretely after intubation, before the start of CPB, and 60 minutes after weaning from CPB when the animal was returned to spontaneous circulation.

Measured and Computed Variables:

The main phase of our study included tracheal intubation with mechanical ventilation and the simulation of CPB or CPB in combination with hypothermic circulatory arrest. The cumulative duration of CPB in all groups was 90 min; next, the animals were weaned from CPB, and a biopsy was taken 1 hour after the animals were returned to spontaneous circulation.

Each animal underwent a bilateral biopsy of the anterolateral and upper and lower pole segments of both kidneys and was euthanized thereafter.

This study aimed to determine the safety of administering 80 ppm nitric oxide and its effectiveness in reducing kidney injury, mitochondrial dysfunction, and regulated cell death in kidneys during experimental cardiopulmonary bypass.

The endpoints of the study are described below.

Mitochondrial disfunction:

- The mtochondrial transmembrane potential ($\Delta\psi$) and the mitochondrial permeability transition pore (mPTP) state, as assessed via the mitochondrial calcium retention capacity (CRC);
- The concentration of adenosine triphosphate (ATP) and lactate in kidney biopsy of experimental animals.

Regulated cell death:

- The concentration of the apoptosis and necroptosis marker tumor necrosis factor α (TNF-α);
- The concentration of the pyroptosis markers nucleotide-binding oligomerization domain (NOD)-like receptor with a pyrin domain 3 (NLRP3) and gasdermin D (GSDMD);
- The concentration of the necroptosis marker receptor-interacting protein kinase 3 (RIPK3).

Kidney injury:

- The concentration of Neutrophil Gelatinase-Associated Lipocalin (uNGAL);
- Diuresis;
- The severity of morphological changes in kidney biopsy specimens;

To assess the safety of the proposed technology, continuous monitoring of the NO_2 concentration in the gas–air mixture delivered via the ventilator circuit and the CPB oxygenator was

carried out. The concentration of methemoglobin (MetHb) and the concentration of the final metabolites of nitric oxide — nitrates and nitrites (NO_2 total, endogenous NO_2 (eNO_2), and NO_3)—in the blood of the experimental animals was also monitored.

The list of safety parameters of the studied method included:

- An increase in MetHb >5%, requiring the discontinuation of NO delivery;
- An increase in the concentration of NO_2 in the inspiratory fraction of the gas–air mixture of more than 2 ppm, requiring the discontinuation of NO delivery.

To monitor the adequacy of anesthesia and CPB during the stages of the study, we assessed ECG data, invasive arterial pressure, central venous pressure, diuresis, body temperature, oxygen saturation, indicators of acid–base status and blood gas composition, and levels of hemoglobin, hematocrit, lactate, and electrolytes.

To achieve the objectives of this study, preclinical, biochemical research methods and an assessment of mitochondrial bioenergetics were used. Pathophysiological substrate and biochemical markers are described below.

Preclinical research:

It was carried out using a sample for plasma–chemical synthesis of nitric oxide for a clinical safety assessment of the proposed technique of perioperative NO conditioning with continuous monitoring of the NO_2 concentration in the gas–air mixture delivered via the circuit of the ventilator and the CPB machine oxygenator. An increase in NO_2 to 2 ppm or more was considered critical; when the mentioned concentration was reached, the delivery of NO was supposed to be discontinued.

Biochemical research:

To assess urinary neutrophil gelatinase-associated lipocalin (uNGAL), urine was sampled directly from a catheter installed into the bladder before the animals were removed from the experiment; the urine was frozen immediately after centrifugation at 1500 g for 10

min. The uNGAL level was determined via an enzyme immunoassay (Hycult Biotech, Netherlands) using a SunRise enzyme immunoassay analyzer (Tecan, USA).

The collection of blood samples for biochemical research was carried out at the following stages: 1—immediately after intubation and before the start of CPB; 2—at the start of CPB; and 3—60 min after weaning the animal from CPB.

NO is a gaseous free radical with a short in vivo half-life (of about a few seconds). The levels of more stable NO metabolites, nitrite (NO_2) and nitrate (NO_3), are used to indirectly detect NO in biological fluids. NO is highly soluble in lipids; it is not stored but is de novo synthesized in L-arginine oxidation and freely diffuses through lipid membranes. In this study, two analysis options were used: measurements of endogenous nitrite (eNO_2), the conversion of nitrate (NO_3) into nitrite (NO_2) using nitrate reductase and the measurement of total nitrite (NO_2 total). To determine the nitrate concentration, the concentration of endogenous nitrite was subtracted from the total nitrite concentration. The concentrations of endogenous nitrite (eNO_2) and nitrate (NO_3), as well as the total concentration of NO metabolites (NOx total, μm/ml), were determined immediately after intubation and before the start of CPB, at the start of CPB, and 60 min after weaning from CPB. For this purpose, 4 ml of blood was collected from a peripheral vein into a test tube containing Ethylenediaminetetraacetic acid (EDTA). The collected samples were centrifuged for 15 min at 3000 g to separate the plasma. The plasma samples were then frozen and stored at -25°C prior to analysis. Plasma NO metabolite levels (nitrites and nitrates) were measured using R&D system kits (R&D Systems NO/Nitrite/Nitrate Kit, nitrate 32–80 μm/L and nitrite 63–165 μm/L) (USA) by the enzymatic colorimetric method. All samples from the same animal were tested with the same set of reagents to avoid intertest variability.

Bioenergetic state of cell research—isolation of mitochondria:

To prevent iatrogenic injury, biomaterial sampling (kidney biopsy) was performed simultaneously with spontaneous circulation at the 60th minute after weaning the animal from machine perfusion. Thus, kidneys were subjected to prolonged CPB and CPB + circulatory arrest (CA). A part of the kidney obtained for the study was placed in a cold buffer solution (2–4°C). The buffer solution itself was composed of 210 mM mannitol (MP Biomedicals, USA), 70 mM sucrose (MP Biomedicals, USA), and 10 mM Hepes (Sigma-Aldrich, USA). These substances were dissolved in deionized water. A solution with pH = 7.4 was used. The pH value was determined using a Sartorius PP-25 pH meter (Sartorius AG, Germany). Isolation of mitochondria from the kidney tissue was carried out by differential centrifugation, as described previously [E13;E14]. To achieve high-quality isolated organelles, all procedures were carried out at a temperature of 4º C in a specially equipped refrigerator. The kidney tissue was minced with scissors into a mushy state in a disposable plastic Petri dish with a diameter of 30 mm. The resulting substance was homogenized using an Ultra-TurraxT10 basic disperser (IKA-Werke, Germany) to obtain a homogeneous substrate in 20 ml of a solution containing sucrose (MP Biomedicals, USA)—70 mmol/L, mannitol (MP Biomedicals, USA)—210 mmol/L, EGTA (ethylene glycol tetraacetic acid) (Sigma-Aldrich, USA)—6 mmol/L, Hepes (Sigma-Aldrich, USA)—10 mmol/L, and BSA (bovine serum albumin fatty acid free) (Sigma-Aldrich, USA)—5 mg/mL. This solution was used at pH=7.4. Homogenates were centrifuged for 10 min at 900 g using a Centrifuge 5810 R (Eppendorf, Germany). The supernatant was collected and passed through a nylon filter and centrifuged again for 10 min at 12000 g. The resulting pellet was resuspended in 20 ml of an isolation buffer (mannitol/sucrose/Hepes) solution supplemented with 0.1 mmol/L EGTA (Sigma-Aldrich, USA) and then centrifuged for 10 min at 12000 g. The final pellet containing the mitochondrial fraction was resuspended in 200 µl of isolation buffer (mannitol/sucrose/Hepes) solution without EGTA and BSA. The protein concentration in the final suspension of mitochondria was determined by the Bradford protein assay [E15].

The method is based on the binding of Coomassie brilliant blue G-250 dye to the amino acid residues of proteins, primarily to arginine, as well as to tryptophan, tyrosine, histidine, and phenylalanine. When binding, the absorption maximum shifts from a 465 nm wavelength (free dye) to 595 nm (bound), when all measurements are performed. The calculation of protein in the test sample was carried out according to the commonly accepted concentrations of standard protein solutions.

Measurement of mitochondrial membrane potential in renal parenchymal biopsy:

The mitochondrial membrane potential was measured using a Shimadzu RF-5301-PC spectrofluorometer (Shimadzu Corporation, Japan), using a tetramethylrhodamine ethyl ester (TMRE; Molecular Probes, Invitrogen, USA), a cationic fluorescent dye, as described previously [E13]. The excitation wavelength was ƛEx=550 nm (ƛEx), and the emission wavelength was ƛEm=575 nm (ƛEm). The reaction was initiated by adding a suspension of mitochondria (2 mg of protein) to a spectrofluorometer cuvette containing 3 ml of a buffer containing 200 mmol/L sucrose (MP Biomedicals, USA), 10 mmol/L Tris-HCl (Sigma-Aldrich, USA), 5 mmol/L KH2PO4 (MP Biomedicals, USA), 0.01 mmol/L EGTA (Sigma-Aldrich, USA), 2.5 mg/mL BSA (Sigma-Aldrich, USA), 5 mmol/L succinate (Sigma-Aldrich, USA), and 40 nmol/L TMRE (MolecularProbes, Invitrogen, USA). The value of the membrane potential was measured by the decrease in fluorescence intensity after the addition of 100 nmol/L FCCP (Sigma-Aldrich, USA) to the incubation medium. The value of the mitochondrial membrane potential was presented as the difference in fluorescence intensity before and after the addition of FCCP (U) per unit mass of protein in the mitochondrial suspension (mg).

Analysis of mitochondrial calcium retention capacity in renal parenchymal biopsy:

The mitochondrial calcium retention capacity (CRC) was assessed using a Ca^{2+}-sensitive fluorescent dye, Calcium Green-5N (MolecularProbes, Invitrogen, USA), using a Shimadzu RF-5301-PC spectrofluorometer (Shimadzu Corporation, Japan) as described

previously [E13]. The excitation wavelength used was ƛEx=555 nm (ƛEx), and the emission wavelength was ƛEm=577 nm (ƛEm). Isolated mitochondria (1 mg protein/mL) were incubated in 3 mL of a buffer containing 200 mmol/L sucrose (MP Biomedicals, USA), 10 mmol/L Tris-HCl (Sigma-Aldrich, USA), 5 mmol/L KH_2PO_4 (MP Biomedicals, USA), 0.01 mmol/L EGTA (Sigma-Aldrich, USA), 2.5 mg/mL BSA (Sigma-Aldrich, USA), and 5 mmol/L succinate (Sigma-Aldrich, USA). The concentration of CalciumGreen-5N was 100 nmol/L. Every 3 minutes, 100 nmol of $CaCl_2$ was added to the mitochondrial incubation medium, and an increase in fluorescence intensity was recorded which then decreased, corresponding to mitochondrial calcium uptake. Portions of the $CaCl_2$ solution were added until a sharp increase in the fluorescence intensity of the CalciumGreen-5N was registered, which corresponded to mitochondrial permeability transition pore (mPTP) opening and Ca^{2+} release from the mitochondria. The Mitochondrial CRC was expressed as the maximum amount of Ca^{2+} accumulated by mitochondria per 1 mg of protein (nmol $CaCl_2$/mg protein).

Determination of ATP in renal parenchymal biopsy:

Sampling of biomaterial (kidney biopsy) was carried out at the same time as spontaneous circulation, at the 60th minute after weaning the animal from machine perfusion. A part of the kidney taken for the study was frozen in liquid nitrogen. The obtained samples were homogenized in liquid nitrogen by adding 2 ml of 3% trichloroacetic acid (TCA) cooled to 2°C.Homogenates were centrifuged for 10 min at 3000 g and 2°C in an SL 16R centrifuge (Thermo Scientific, USA) as described previously [E13]. The supernatant was taken, neutralized with 1M Trizma base (Sigma-Aldrich, USA), and the sample volume was adjusted to 2 ml with deionized water. ATP was determined using the ATP Bioluminescent Assay Kit (Sigma-Aldrich, USA) on a Lucy-2 luminometer (Anthos Labtec Instruments, Austria). The principle of this analysis was based on the following reactions:

ATP + Luciferin → (luciferase enzyme) Adenyl-luciferin + PPi (1);

Adenyl-luciferin + O2 → Oxyluciferin + AMP + CO2 + light (2).

The first reaction is reversible, and its equilibrium is shifted to the right; the second reaction is irreversible. Thus, the amount of ATP in the initial sample is a limiting factor of the reaction, and the amount of emitted light is directly proportional to the ATP content in the solution.

Determination of lactate concentration in renal parenchymal biopsy:

Biomaterial (kidney biopsy) was sampled simultaneously with spontaneous circulation at the 60th minute after weaning the animal from machine perfusion. A part of the kidney taken for the study was frozen in liquid nitrogen. The obtained samples were homogenized in liquid nitrogen by adding 2 mL of 3% trichloroacetic acid (TCA) cooled to 2°C. Homogenates were centrifuged for 10 minutes at 3000 g and 2°C in an SL 16R centrifuge (Thermo Scientific, USA). The supernatant was taken, neutralized with 1M Trizma base (Sigma-Aldrich, USA), and the sample volume was adjusted to 2 ml with deionized water. Lactate concentration was determined by the enzyme immunoassay using the L-Lactate Assay Kit (Sigma-Aldrich, USA) by a multifunctional microplate reader Infinite 200 (Tecan, Austria). Color intensity is proportional to the concentration of lactate in the sample. The principle of this assay is based on lactate oxidation catalyzed by lactate dehydrogenase. The NADH produced during this reaction reduces a formazan reagent, and the color intensity of the resulting solution is proportional to the lactate concentration in the sample.

Receptor-Interacting Protein Kinase 3 (RIPK3) in renal parenchymal biopsy:

The sampling of biomaterials (kidney biopsy) was carried out at the same time with spontaneous circulation, at the 60th minute after weaning from machine perfusion. A part of the kidney used for the study was frozen in liquid nitrogen. The obtained samples were homogenized in liquid nitrogen by adding 500 µl of phosphate buffered saline (PBS; pH 7.0) cooled to 2°C. Several freeze–thaw cycles were used to disrupt cell membranes. Homogenates

were centrifuged for 15 minutes at 1500 g and 2°C in an SL 16R centrifuge (Thermo Scientific, USA). The supernatant was collected in 100 μl Eppendorf tubes and frozen at -80°C. RIPK3 was determined by an enzyme immunoassay using a Sheep Receptor Interacting Serine Threonine Kinase 3 (RIPK3) ELISA kit (Bluegene Biotech, China) on an Infinite 200 multifunctional microplate reader (Tecan, Austria). The principle of this assay is based on a competitive ELISA method using a polyclonal antibody to RIPK3 and a horseradish peroxidase conjugate. Samples and a buffer were incubated with the enzyme conjugate in a plate for 1 hour. The unbound material was then removed by multiple washes (at least five). Next, a chromogenic substrate was added to each well. The enzyme interacts with the substrate, producing a blue color. The enzymatic reaction was terminated by adding the Stop solution. The intensity of the color in the wells was inversely proportional to the concentration of RIPK3 since the RIPK3 from the samples competed with enzyme-bound RIPK3 for RIPK3 antibody binding sites. Since the number of binding sites was limited, the more sites there were occupied by RIPK3 from a sample, the fewer sites there were occupied by RIPK3 with horseradish peroxidase. The concentration of RIPK3 in the samples is measured according to the standard (calibration) curve.

NLR pyrin domain-containing protein 3 (NLRP3) in renal parenchymal biopsy:

The sampling of biomaterial (kidney biopsy) was carried out at the same time as spontaneous circulation, at the 60th minute after weaning the animal from machine perfusion. A part of the kidney taken for study was frozen in liquid nitrogen. The obtained samples were homogenized in liquid nitrogen by adding 500 μl of phosphate buffer (PBS, pH 7.0) cooled to 2°C. Several freeze–thaw cycles were used to disrupt cell membranes. Homogenates were centrifuged for 15 minutes at 1500 g and 2°C in an SL 16R centrifuge (Thermo Scientific, USA). The supernatant was collected in 100 μl Eppendorf tubes and frozen at -80°C. NLRP3 was determined by an enzyme immunoassay using a Sheep NLR Pyrin Domain Containing

Proteins 3 (NLRP3) Elisa kit (Bluegene Biotech, China) on an Infinite 200 multifunctional microplate reader (Tecan, Austria). This kit is based on a competitive ELISA method using a polyclonal antibody to NLRP3 and NLRP3 horseradish peroxidase conjugate. The samples and buffer were incubated with the enzyme conjugate in the plate for 1 hour. The unbound material was then removed by multiple washes (at least five). A chromogenic substrate was added to each well. The enzyme interacted with the substrate, producing a blue color. The enzymatic reaction was terminated by adding the Stop solution. The intensity of the color in the wells was inversely proportional to the concentration of NLRP3 since the NLRP3 from the samples competed with the enzyme-bound NLRP3 for NLRP3 antibody binding sites. Since the number of binding sites is limited, the more sites there were occupied by NLRP3 from a sample, the fewer sites there were occupied by NLRP3 with horseradish peroxidase. The concentration of NLRP3 in the samples was measured according to the standard (calibration) curve.

Gasdermin D (GSDMD) in renal parenchymal biopsy:

The sampling of biomaterial (kidney biopsy) was carried out at the same time as spontaneous circulation, at the 60th minute after weaning the animal from machine perfusion. A part of the kidney taken for the study was frozen in liquid nitrogen. The obtained samples were homogenized in liquid nitrogen by adding 500 µl of phosphate buffer (PBS, pH 7.0) cooled to 2°C. Several freeze–thaw cycles were used to disrupt cell membranes. Homogenates were centrifuged for 15 minutes at 1500 g and 2°C in an SL 16R centrifuge (Thermo Scientific, USA). The supernatant was collected in 100 µl Eppendorf tubes and frozen at -80°C. GSDMD was determined by an enzyme immunoassay using a Sheep Gasdermin D (GSDMD) Elisa kit (Bluegene Biotech, China) on an Infinite 200 multifunctional microplate reader (Tecan, Austria). The kit is based on a competitive ELISA method using a polyclonal antibody to GSDMD and GSDMD horseradish peroxidase conjugate. The samples and buffer were incubated with the enzyme conjugate in the plate for 1 hour. The unbound material was then

removed by multiple washes (at least five). A chromogenic substrate was added to each well. The enzyme interacted with the substrate, producing a blue color. The enzymatic reaction was terminated by adding the Stop solution. The intensity of the color in the wells was inversely proportional to the concentration of GSDMD since the GSDMD from the samples competes with the enzyme-bound GSDMD for GSDMD antibody binding sites. Since the number of binding sites was limited, the more sites there were occupied by GSDMD from a sample, the fewer sites there were occupied by GSDMD with horseradish peroxidase. The concentration of GSDMD in the samples was measured according to the standard (calibration) curve.

Tumor necrosis factor α (TNF-α) in renal parenchymal biopsy:

The sampling of biomaterial (kidney biopsy) was carried out at the same time as spontaneous circulation, at the 60th minute after weaning the animal from machine perfusion. A part of the kidney taken for the study was frozen in liquid nitrogen. The obtained samples were homogenized in liquid nitrogen by adding 500 µl of phosphate buffer (PBS, pH 7.0) cooled to 2°C. Several freeze–thaw cycles were used to disrupt cell membranes. Homogenate were centrifuged for 15 minutes at 1500 g and 2°C in an SL 16R centrifuge (Thermo Scientific, USA). The supernatant was collected in 100 µl Eppendorf tubes and frozen at -80°C. TNF-α was determined by an enzyme immunoassay using a Sheep Tumor Necrosis Factor Alpha (TNF Alpha) Elisa kit (Bluegene Biotech, China) on an Infinite 200 multifunctional microplate reader (Tecan, Austria). The kit is based on a competitive ELISA method using a polyclonal antibody to TNF-α and TNF-α horseradish peroxidase conjugate. The samples and buffer were incubated with the enzyme conjugate in the plate for 1 hour. The unbound material was then removed by multiple washes (at least five). A chromogenic substrate was added to each well. The enzyme interacted with the substrate, producing a blue color. The enzymatic reaction was terminated by adding the Stop solution. The intensity of the color in the wells was inversely proportional to the concentration of TNF-α since the TNF-α from samples competes with the enzyme-bound

TNF-α for TNF-α antibody binding sites. Since the number of binding sites was limited, the more sites there were occupied by TNF-α from a sample, the fewer sites there were occupied by TNF-α with horseradish peroxidase. The concentration of TNF-α in the samples was measured according to the standard (calibration) curve.

Histological examination of renal parenchymal biopsy:

Tissue samples from the experimental animals were placed in a 10% buffered (pH 7.4) formalin solution (BioVitrum, Russia). After 12-24 hours of fixation in formalin, the tissue samples were washed under running water, dehydrated with IsoPrep solutions of increasing concentration (BioVitrum, Russia) in eight procedures, impregnated, and embedded into Histomix paraffin mixture (BioVitrum, Russia). Paraffin sections 5 μm thick were prepared on an automatic microtome (HM355S, Thermo Fisher Scientific, China).

The sections were deparaffinized in o-xylene (EKOS-1, Russia), rehydrated in absolute ethyl alcohol and distilled water via three procedures, and stained in accordance with the recommendations of the dye manufacturer. The stained preparations were dehydrated in absolute ethyl alcohol, cleared in o-xylene (EKOS-1, Russia), and mounted in Canada balsam (Pancreac, Spain).

The deparaffinized sections of the internal organs of the experimental animals were stained with Gill's hematoxylin (BioVitrum, Russia) and eosin (BioVitrum, Russia).

Histology slides were examined using an Axioscope 40 light microscope (Zeiss, Germany). Photos of the histology slides were obtained using a Canon G5 digital camera (Canon, Japan) with AxioVision 4.3 (Zeiss, Germany).

In the histological preparations, the cross-sectional area of the renal corpuscle (Src), the cross-sectional area of the glomerulus (Sg), the area of the proximal convoluted tubule (Spct), and the lumen area of the proximal convoluted tubule (Slpct) were measured. The glomerular–capsular index (GCI) and lumen–epithelial index (LEI) were calculated.

The GCI was calculated using the following formula: GCI=Sg/Src-Sg.

The LEI was calculated using the following formula: LEI=Slpct/Spct-Slpct.

Justifying the choice of Endpoints:

Nitric oxide homeostasis:

In the intraoperative period, cardiac surgery patients are exposed to complex pathological factors, including surgical trauma, anesthesia, and extracorporeal circulation. Postperfusion syndrome after cardiac surgery with CPB includes the development of intravascular hemolysis [E16;E17]. Hemolysis can develop as a result of cell injury when aspirating a surgical wound, line occlusion with a roller pump during machine perfusion, the development of turbulent blood flow in the oxygenator and constriction areas of the extracorporeal circuit, as well as cell separation with Cell-Saver devices. Reduced iron (Fe^{2+}) released from the hemoglobin molecule can participate in reactions with organic and inorganic oxygen radicals and stimulate lipid peroxidation and the activation of the formation of hydroxyl radicals with subsequent tissue injuries, exerting a direct nephrotoxic effect and contributing to oxidative damage to the renal tubular epithelium [E17;E18]. In about 25% of cases, free hemoglobin and reduced iron generation after CPB exceed serum iron-binding capacity, which greatly aggravates reperfusion injury [E19;E20;E21]. Free hemoglobin binds NO produced by the endothelium, which leads to a decrease in the bioavailability of this regulatory molecule and microcirculatory disorders [E22]. This phenomenon may be associated with a decrease in organ perfusion and oxygen delivery to the renal medulla; regional ischemia leads to tubular injury with aberrations in renal function in the postoperative period [E23;E24]. NO sequestration leads to systemic damage to splanchnic organs [E25]. Massive intravascular hemolysis and hemoglobinemia induce the development of multiple-organ damage with intravascular coagulation and the formation of renal, respiratory and, ultimately, multiple-organ

failure [E26;E27;E28]. In some cases (with massive transfusion, operations with deep hypothermia, and prolonged CPB), the concentration of free hemoglobin can reach 1000 mg/dL [E29]; however, concomitant injuries in splanchnic organs and, in particular, in the kidneys, can develop even with low-level intravascular hemolysis, and peak concentrations of free hemoglobin correlate with postoperative kidney injury, while hemoglobin in urine may not yet be determined [E28;E30]. In the postoperative period following cardiac surgery, multiple organ complications, including renal ones, are based on systemic microcirculatory disorders. The pathogenic mechanism in this case is the sequestration of nitric oxide, which is responsible for the mechanisms of vascular wall relaxation, by free hemoglobin. The reaction is extremely fast (about 10 ms), with deoxyhemoglobin reversibly binding to NO to form nitrosyl hemoglobin and oxyhemoglobin irreversibly forming nitrate and methemoglobin [E31]. A free hemoglobin concentration as low as 10 mg/dL can inhibit vasodilation in vivo [E32;E33;E34]. Thus, disorders of endogenous NO homeostasis, including those associated with CPB-induced hemolysis, are an extremely important trigger in the pathogenesis of postoperative complications in cardiac surgery patients.

Mitochondrial dysfunction and energy substrates:

The main source of energy in most cells, with the exception of erythrocytes, are mitochondria. Mitochondria are the main source of energy in the form of ATP molecules, and they are directly involved in maintaining ion homeostasis in the cell, generate reactive oxygen species, and can also be triggers of apoptosis. In the cytoplasm, during glycolysis, the breakdown of one glucose molecule into lactate produces four ATP molecules [E35]. Eleven ATP molecules are formed from the oxidation of one acetyl CoA molecule in the Krebs cycle, [E35]. According to Mitchell's chemiosmotic theory, mitochondria convert the chemical energy released in oxidation of acetyl CoA into the electrical potential on the inner mitochondrial membrane ($\Delta\psi$). ATP synthase converts electrical energy into chemical energy, namely, it

synthesizes ATP [E36], which is transported by ADP/ATP translocase from the mitochondria to the cytoplasm [E37]. Therefore, the greater the $\Delta\psi$, the more efficiently mitochondria synthesize ATP. In case of oxidative phosphorylation uncoupling, the $\Delta\psi$ drops to zero, and ATP synthesis in the mitochondria stops. An important regulator of the functional state of the cell and mitochondria is the mitochondrial permeability transition pore (mPTP), a protein complex built into cell membranes [E38;E39]. According to A.P. Halestrap, the mPTP is "the major player" which can make a cell live or die. [E38]. The opening of mPTPs leads to a drop in the $\Delta\psi$, and ATP synthesis in mitochondria stops, which ultimately leads to the death of cells with a high demand for ATP, for instance, kidney cells [E40]. Even if not all mPTPs in mitochondria are open and $\Delta\psi$ does not decrease to zero, the cell can die as a result of apoptosis, since the opening of this pore leads to the release of cytochrome C and AIF (apoptosis-inducing factor) protein from mitochondria [E39]. These proteins are triggers for programmed cell death [E39].

CPB is associated with a number of damaging factors: ischemia–reperfusion, the activation of systemic inflammation, the formation of reactive oxygen species, oxidative stress caused by the persistence of extracellular hemoglobin, etc. Mediators of ischemia–reperfusion organ injury are the activation of the complement system and leukocytes, an increase in the concentration of reactive oxygen species, oxygen, reduced efficiency of mitochondrial oxidative phosphorylation, endothelial dysfunction, and, ultimately, the triggering of apoptosis, necrosis, or autophagy. Due to damaging factors, in particular, during ischemia–reperfusion in renal parenchyma, the lack of oxygen supply disrupts the mitochondrial electron transport chain function, resulting in a decrease in the ATP concentration in kidney tissue. Mitochondrial ATP synthase, which normally pumps protons from the intermembrane space to the matrix and synthesizes ATP molecules, begins to work in the opposite direction during ischemia. This enzyme consumes 50–80% of ATP for pumping protons from the matrix to the intermembrane

space, maintaining the mitochondrial membrane potential (Δψ). The activation of anaerobic glycolysis, lactic acidosis, and ATP hydrolysis lead to a significant decrease in intracellular pH. The accumulation of [H^+] activates the Na^+/H^+ antiporter, which leads to an increase in the concentration of intracellular sodium ions ([Na^+]$_i$). The accumulation of [Na^+]$_i$ is also due to a decrease in the activity of Na^+/K^+-ATP because of a decrease in the intracellular concentration of ATP. An increase in the concentration of [Na^+]$_i$ leads to the reverse mode of the Na^+/Ca^{2+} antiporter and promotes an increase in the concentration of [Ca^{2+}]$_i$ inside the cell [E41]. Excessive calcium accumulation in the cytoplasm was observed during ischemia–reperfusion. This calcium is accumulated by mitochondria. The transport of calcium ions into mitochondria is carried out by a Ca^{2+} uniporter due to the energy of the Δψ, i.e., without ATP synthesis [E41]. In addition to this mechanism, the accumulation of calcium by mitochondria during ischemia occurs due to the reverse mode of the mitochondrial Na^+/Ca^{2+} exchanger. The uptake of excessive Ca^{2+} by mitochondria makes it possible to compensate for the cytoplasmic Ca^{2+} overload. At the same time, it is known that the concentration of [Ca^{2+}]$_m$ during reperfusion reaches a maximum of 0.3–0.4 μM, a threshold above which its excess exacerbates ischemia–reperfusion injury. This threshold value is also due to mPTPs that are sensitive to [Ca^{2+}]$_m$. The mPTP is embedded in the inner and outer mitochondrial membranes [E38;E40;E39]. It provides membrane permeability for ions and proteins weighing up to 1.5 kDa and is closed under physiological conditions. A necessary condition for mPTP opening is an increased concentration of calcium ions in the mitochondria and the excessive production of reactive oxygen species (ROS), which is observed during reperfusion. The state of the mPTP is a key moment in triggering cell death when it can no longer resist ischemia. The opening of the mPTP and a decrease in the Δψ are events in which cell death becomes irreversible. The opening of the mPTP leads to mitochondrial swelling, outer mitochondrial membrane rupture, the inhibition of mitochondrial function, as well as the release of cytochrome C and pro-apoptotic

protein apoptosis-inducing factor into the cytoplasm of cells and triggering apoptosis or necrosis [E38;E40;E39;E41]. Cell death depends on the energy state of the cell since apoptosis is an energy-dependent process and requires ATP molecules in the cell.

Thus, metabolic changes in organs and tissues during ischemia and reperfusion lead to mitochondrial damage and the disruption of the functional state of mitochondria.

Ischemia–reperfusion is the cause of an imbalance between the supply and demand of organs for oxygen and energy substrates. A decrease in oxygen supply causes disorders in oxidative phosphorylation and, accordingly, leads to a decrease in ATP synthesis in mitochondria in various organs [E42]. At the same time, ATP deficiency depends on the degree and duration of ischemia. The lack of oxygen and energy substrates promotes the activation of anaerobic glycolysis. The activation of glycolysis leads to the accumulation of lactate and protons in the cytoplasm [E42]. Fatty acid β-oxidation inhibition leads to the accumulation of its metabolites, in particular acyl-CoA, which cause damage to mitochondrial membranes, disrupt the mitochondrial cristae, and cause the dysfunction of organelles [E42].

Thus, the value of the $\Delta\psi$ determines the efficiency of ATP synthesis in mitochondria, and cell survival under unfavorable conditions depends on the mPTP state and mitochondrial Ca^{2+} retention capacity. Measuring ATP and lactate concentrations determines the functional state of mitochondria after ischemia, as well as any drugs used to protect cells from hypoxia.

Regulated cell death:

Mitochondrial dysfunction in ischemia and reperfusion, as well as the excessive accumulation of calcium ions, leads to enzyme activation that triggers a cascade of reactions leading to various types of regulated cell death (necroptosis, pyroptosis, and apoptosis). The severity of target organ damage, as well as the effectiveness of organoprotective methods, can be assessed by the activation of the signaling pathways of necroptosis, pyroptosis, and apoptosis in tissues [E41;E43;E44; E45].

Apoptotic cell death occurs against a background of maintained cell membrane integrity and therefore is not accompanied by an inflammatory response [E41]. Apoptotic cell death is triggered by both extrinsic and intrinsic signals. In the extrinsic pathway of apoptosis, an important role is played by the activation of receptors for tumor necrosis factor-α and Fas receptors. The final effect is achieved by caspase-8 and caspase-3 activation, which directly destroys the cell during apoptosis. The triggering of the intrinsic pathway of apoptosis is associated with the opening of mPTP and the Bax and Bak pores. The opening of these pores leads to a drop in the $\Delta\psi$, the release of proapoptotic proteins from the mitochondria, as well as the entry of ions and water into the mitochondria, their swelling, and mitochondrial membrane rupture [E38;E40;E39;E41]. Further activation of effector caspase-3 and -7 leads to the destruction of DNA and the cytoskeleton. As a result, chromatin condensation and apoptotic cell shrinkage occur. mPTP-induced apoptosis is believed to be the main mechanism of cell death during reperfusion [E41].

Necroptosis can be triggered in cases where apoptosis is impossible for one reason or another [E43]. Unlike apoptosis, necroptosis is accompanied by the release of cell content into the external environment and causes a strong response from the immune system. The triggering of necroptosis is also associated with the activation of tumor necrosis factor receptors, which, in turn, activate the RIPK1 and RIPK3. The process of necroptosis is associated mainly with events occurring in the cell in ischemia and reperfusion [E43].

The main distinction of pyroptosis is an obligatory inflammatory component [E44;E45]. A key role in pyroptosis is played by caspase-1 (not involved in apoptosis), which converts the precursors of the proinflammatory cytokines IL-1β and IL-18 into their active forms. Activated cytokines increase leukocyte migration into tissues involving other cytokines and chemokines in the process, which contributes to an even greater inflammatory process.

Most researchers are inclined to adopt the point of view that the activation of pyroptosis and necroptosis aggravates ischemic and reperfusion injuries due to the formation of inflammation and intracellular structures that damage the cell membrane [E43;E44;E45]. One of the main inflammatory cytokines is TNF-α, which causes the activation of apoptosis and necroptosis [E43]. In turn, the formation of necroptosis is also associated with the activation of receptor-interacting protein kinase-3 (RIPK3), which could be involved both in apoptosis and necroptosis [E43].

In the suppression of inflammation (TNF-α), the necroptosis predictors RIPK3 and pyroptosis NLRP3 and Gasdermin D are favorable factors for reducing ischemic and reperfusion injury. Therefore, the levels of pyroptosis, apoptosis, and necroptosis markers (NLRP3, Gasdermin D, RIPK3, and TNF-α) can determine the effectiveness of protective drugs used to reduce organ damage as a result of exposure to prolonged ischemia and/or hypoxia. At the same time, the overexpression of TNF-α is responsible for the initiation of apoptosis, the overexpression of RIPK3 is responsible for the initiation of necroptosis, and the overexpression of NLRP3 and Gasdermin D (GSDMD) is responsible for the initiation of pyroptosis.

Statistical Analysis:

Reporting any deviations from the original statistical plan:

Any deviations from the original statistical plan will be detailed and justified in the relevant protocol amendments, as well as the final report of this experimental study.

Selection procedure:

To test the study's primary hypothesis, the data set was analyzed according to the protocol to provide Intention-to-treat (ITT) analysis—ITT set. A similar approach was used to test secondary hypotheses about the effectiveness of NO therapy for secondary endpoints.

To assess safety parameters of NO therapy, including adverse events, a Per protocol set (PPS) was used.

General strategy and statistical analysis methods:

Statistical analysis was performed in Statistics 10.0 and Jamovi. The normality of the distribution of quantitative variables was tested using the Shapiro–Wilk test. If the variables had a normal distribution, they were described by their mean values and standard deviations, Ms ± SDs; otherwise, they were described using medians and interquartile intervals, Me (Q1;Q3). Differences in total and exploratory quantitative variables in independent groups 1-4 were analyzed using a one-way analysis of variance (one-way ANOVA) and Student's t-test (Welch's t-test with heterogeneity of variance in groups) for independent groups (independent samples t-test) with normal distribution of the variable in all compared groups, or using the Kruskal–Wallis test and Mann–Whitney U test otherwise. If the variable had a normal distribution at all three stages of measurement, the identification of statistically significant dynamic differences in variables at these stages in groups was carried out using a repeated-measures MANOVA with Mauchly's sphericity test using the Greenhouse–Geisser correction or the Huyne–Feldt correction for a lack of sphericity. For post hoc comparisons, Student's t-test for paired samples (paired t-test) was used with Bonferroni's p-adjustment for multiple comparisons.

Otherwise, Friedman's test with post hoc comparisons via Wilcoxon's test and adjustments for multiple comparisons were used to compare variables at three stages. In cases where frequency statistics (the p-value) did not allow for the rejection of the null hypothesis of the equality of means in the compared groups, we applied the Bayes factor.

The threshold significance level for testing the hypotheses was $p=0.05$.

Sample size:

Twenty-four sheep of the Altai breed were divided into four groups of six each, as described in the manuscript. The reason for the relatively small sample size was because our study was the first to evaluate mitochondrial dysfunction in vivo as a fundamental basis for the

organoprotective effects of NO in cardiac surgery. Our goal was also to collect basic evidence for the safety and efficacy of plasma–chemical NO synthesis for the implementation of therapeutic approaches in a preclinical experimental project.

The sample size was also influenced by ethical considerations, financial costs, the need for great human resources, and the logistics of conducting experiments involving large animals with CPB. To confirm our hypothesis, it was decided to limit the sample size to the minimum possible number, n = 6, acceptable for the vast majority of such experiments.

Additional aspects:

Ethics:

This study was conducted in accordance with international standards for the humane care and use of laboratory animals and was governed by Directive 2010/63/EU of the European Parliament and of the Council of September 22, 2010 on the protection of animals used for scientific purposes. Prior to the start of this experimental study, information about this study, including the study protocol, information about the experience of the researchers in their relevant specialties and their experience in conducting experimental studies, was submitted to the Institutional Review Board (IRB), Biomedical Ethics Committee of Cardiology Research Institute, Tomsk NRMC, for ethical review. This study only commenced upon receiving the approval of the experimental study from the IRB, which also controlled the observance of ethical standards in the conduct of this study. The researchers are responsible for all matters related to the welfare of the animals, including their maintenance and care, throughout the entire period of the study approved by IRB. The research leader is solely responsible for ensuring that all individuals involved in the project understand and accept responsibility for the care and use of animals in research. Investigators were required to report adverse events in laboratory animals within 24 hours of the event. Researchers were required to take prompt action after

adverse events to alleviate the pain and suffering of the animals, and if necessary, the animals were to be withdrawn from the study in a humane manner without delay. Final reports were submitted upon completion of the experimental study to the IRB.

Data and record management:

Baseline, procedural, and intraoperative data were collected and archived. All data were stored electronically (database) using appropriate, certified software with a quality management system. All paper forms are stored in a secure, locked filing cabinet with limited access. Animals were identified in the database using a unique number obtained during randomization.

Role of funding source:

The Ministry of Science and Higher Education of the Russian Federation supported this work. The funding source had no involvement in study design, collection, analysis, and interpretation of data, writing of the report, and the decision to submit the paper for publication. All authors confirm that they had full access to all the data in the study and accept responsibility during the submission.

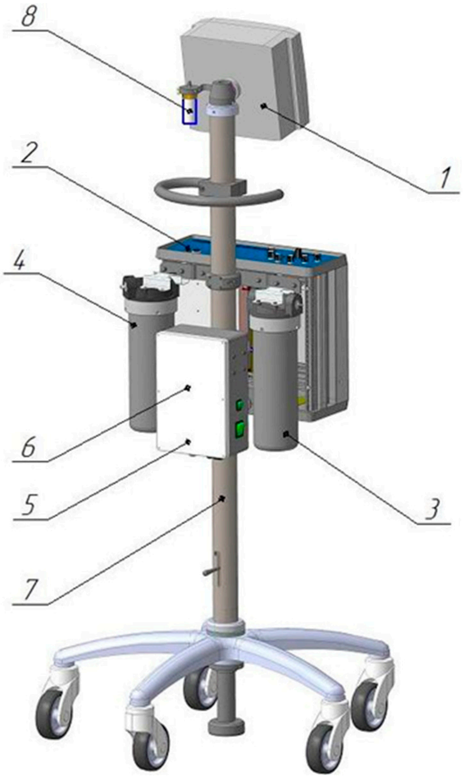

Figure S1. Device for nitric oxide therapy: —NO- and NO_2-monitoring unit; 2—generator; 3—neutralizer; 4 – purification unit; 5—electric unit; 6—air supply unit; 7—rack with base; and 8—moisture separator.

Reference

E1. Göthberg S, Edberg KE. Inhaled nitric oxide to newborns and infants after congenital heart surgery on cardiopulmonary bypass. A dose-response study. Scand Cardiovasc J 2000;34(2):154-8.

E2. Germann P, Braschi A, Della Rocca G, Dinh-Xuan AT, Falke K, Frostell C, et al. Inhaled nitric oxide therapy in adults: European expert recommendations. Intensive Care Med 2005;31(8):1029-41.

E3. Piknova B, Gladwin MT, Schechter AN, Hogg N. Electron paramagnetic resonance analysis of nitrosylhemoglobin in humans during NO inhalation. J Biol Chem 2005;280(49):40583-8.

E4. Gladwin MT, Schechter AN, Shelhamer JH, Pannell LK, Conway DA, Hrinczenko BW, et al. Inhaled nitric oxide augments nitric oxide transport on sickle cell hemoglobin without affecting oxygen affinity. J Clin Invest 1999;104(7):937-45.

E5. Nagasaka Y, Fernandez BO, Steinbicker AU, Spagnolli E, Malhotra R, Bloch DB, et al. Pharmacological preconditioning with inhaled nitric oxide (NO): Organ-specific differences in the lifetime of blood and tissue NO metabolites. Nitric Oxide 2018;80:52-60

E6. Vermeulen Windsant IC, Snoeijs MG, Hanssen SJ, Altintas S, Heijmans JH, Koeppel TA, et al. Hemolysis is associated with acute kidney injury during major aortic surgery. Kidney Int 2010;77(10):913-20.

E7. Vermeulen Windsant IC, de Wit NC, Sertorio JT, van Bijnen AA, Ganushchak YM, Heijmans JH, et al. Hemolysis during cardiac surgery is associated with increased intravascular nitric oxide consumption and perioperative kidney and intestinal tissue damage. Front Physiol 2014;5:340.

E8. Chang WL, Corate LM, Sinclair JM, van der Heyde HC. Continuous inhaled nitric oxide therapy in a case of sickle cell disease with multiorgan involvement. J Investig Med 2008;56(8):1023-7.

E9. Kamenshchikov NO, Anfinogenova YJ, Kozlov BN, Svirko YS, Pekarskiy SE, Evtushenko VV, et al. Nitric oxide delivery during cardiopulmonary bypass reduces acute kidney injury: a randomized trial. *J Thorac Cardiovasc Surg* 2022;163(4):1393-1403.

E10. Troncy E, Francoeur M, Salazkin I, Yang F, Charbonneau M, Leclerc G, et al. Extra-pulmonary effects of inhaled nitric oxide in swine with and without phenylephrine. Br J Anaesth 1997;79(5):631-40.

E11. Berra L, Pinciroli R, Stowell CP, Wang L, Yu B, Fernandez BO, et al. Autologous transfusion of stored red blood cells increases pulmonary artery pressure. Am J Respir Crit Care Med 2014;190(7):800-7.

E12. Lei C, Berra L, Rezoagli E, Yu B, Dong H, Yu S, et al. Nitric oxide decreases acute kidney injury and stage 3 chronic kidney disease after cardiac surgery. Am J Respir Crit Care Med 2018;198(10):1279-1287.

E13. Prokudina ES, Naryzhnaya NV, Mukhomedzyanov AV, Gorbunov AS, Zhang Y, Yaggi AS, et al. Effect of chronic continuous normobaric hypoxia on functional state of cardiac mitochondria and tolerance of isolated rat heart to ischemia and reperfusion: role of μ and delta2 opioid receptors. Physiol Res 2019;68(6):909-920.

E14. Prokudina ES, Naryzhnaya NV, Nesterov EA, Tsibulnikov SY, Maslov LN. Continuous normobaric hypoxia improved cardiac bioenergetics after ischemia/reperfusion: role of opioid receptors. Bull Exp Biol Med 2020;169(1):13-17.

E15. Bradford MM. A rapid and sensitive method for the quantitation of microgram quantities of protein utilizing the principle of protein-dye binding. Anal Biochem 1976; 72(1-2):248-254.

E16. Vercaemst L. Hemolysis in cardiac surgery patients undergoing cardiopulmonary bypass: a review in search of a treatment algorithm. J Extra Corpor Technol 2008;40(4):257.

E17. Wright G. Haemolysis during cardiopulmonary bypass: update. Perfusion 2001;16(5):345-351.

E18. Flaherty J. T., Weisfeldt M. L. Reperfusion injury. Free Radic Biol Med 1988;5(5-6):409-419.

E19. Baliga R, Ueda N, Walker PD, Shah SV. Oxidant mechanisms in toxic acute renal failure. Am J Kidney Dis 1997;29(3):465-77.

E20. Pepper JR, Mumby S, Gutteridge JMC. Sequential oxidative damage, and changes in iron-binding and iron-oxidising plasma antioxidants during cardiopulmonary bypass surgery. Free Radic Res 1994;21(6):377-385.

E21. Pepper JR, Mumby S, Gutteridge JMC. Blood cardioplegia increases plasma iron overload and thiol levels during cardiopulmonary bypass. Ann Thorac Surg 1995;60(6):1735-1740.

E22. Reiter CD, Wang X, Tanus-Santos JE, Hogg N, Cannon RO 3rd, Schechter AN, et al. Cell-free hemoglobin limits nitric oxide bioavailability in sickle-cell disease. Nat Med 2002;8(12):1383-1389.

E23. Rother RP, Bell L, Hillmen P, Gladwin MT. The clinical sequelae of intravascular hemolysis and extracellular plasma hemoglobin: a novel mechanism of human disease. JAMA 2005;293(13):1653-1662.

E24. Windsant ICV, Snoeijs MG, Hanssen SJ, Altintas S, Heijmans JH, Koeppel TA, et al. Hemolysis is associated with acute kidney injury during major aortic surgery. Kidney Int 2010;77(10):913-920.

E25. Windsant ICV, de Wit NC, Sertorio JT, van Bijnen AA, Ganushchak YM, Heijmans JH, et al. Hemolysis during cardiac surgery is associated with increased intravascular nitric oxide consumption and perioperative kidney and intestinal tissue damage. Front Physiol 2014;5:340.

E26. Haase M, Haase-Fielitz A, Bellomo R. Cardiopulmonary bypass, hemolysis, free iron, acute kidney injury and the impact of bicarbonate. Contrib Nephrol. 2010;165:28-32.

E27. Jeffers A, Gladwin MT, Kim-Shapiro DB. Computation of plasma hemoglobin nitric oxide scavenging in hemolytic anemias. Free Radic Biol Med 2006;41(10):1557-65.

E28. Kato GJ, Taylor JG 6th. Pleiotropic effects of intravascular haemolysis on vascular homeostasis. Br J Haematol 2010;148(5):690-701.

E29. Ratnam S, Mookerjea S. The regulation of superoxide generation and nitric oxide synthesis by C-reactive protein. Immunology 1998;94(4):560-568.

E30. Davis CL, Kausz AT, Zager RA, Kharasch ED, Cochran RP. Acute renal failure after cardiopulmonary bypass in related to decreased serum ferritin levels. J Am Soc Nephrol 1999;10(11):2396-2402.

E31. Olson JS, Foley EW, Rogge C, Tsai AL, Doyle MP, Lemon DD. No scavenging and the hypertensive effect of hemoglobin-based blood substitutes. Free Radic Biol Med 2004;36(6):685-697.

E32. Meyer C, Heiss C, Drexhage C, Kehmeier ES, Balzer J, Mühlfeld A, et al. Hemodialysis-induced release of hemoglobin limits nitric oxide bioavailability and impairs vascular function. J Am Coll Cardiol 2010;55(5):454-459.

E33. Nakai K, Ohta T, Sakuma I, Akama K, Kobayashi Y, Tokuyama S, et al. Inhibition of endothelium-dependent relaxation by hemoglobin in rabbit aortic strips: comparison between acellular hemoglobin derivatives and cellular hemoglobins. J Cardiovasc Pharmacol 1996;28(1):115-123.

E34. Pohl U, Lamontagne D. Impaired tissue perfusion after inhibition of endothelium-derived nitric oxide. Basic Res Cardiol 1991;86:97-105.

E35. Lehninger A. Biochemistry: The Molecular Basis of Cell Structure and Function. Worth Publishers;1970.

E36. Mitchell P, Moyle J. Translocation of some anions cations and acids in rat liver mitochondria. Eur J Biochem 1969;9(2):149-155.

E37. Nury H, Dahout-Gonzalez C, Trézéguet V, Lauquin GJ, Brandolin G, Pebay-Peyroula E. Relations between structure and function of the mitochondrial ADP/ATP carrier. Annu Rev Biochem 2006;75:713-741.

E38. Halestrap AP. A pore way to die: the role of mitochondria in reperfusion injury and cardioprotection. Biochem Soc Trans 2010;38(4):841-860.

E39. Kroemer G, Galluzzi L, Brenner C. Mitochondrial membrane permeabilization in cell death. Physiol Rev 2007;87(1):99-163.

E40. Naryzhnaya NV, Maslov LN, Oeltgen PR. Pharmacology of mitochondrial permeability transition pore inhibitors. Drug Dev Res 2019;80(8):1013-1030.

E41. Maslov LN, Popov SV, Mukhomedzyanov AV, Naryzhnaya NV, Voronkov NS, Ryabov VV, et al. Reperfusion cardiac injury: receptors and the signaling mechanisms. Curr Cardiol Rev 2022;18(5):63-79.

E42. Frangogiannis NG. Pathophysiology of myocardial infarction. Compr Physiol 2015; 5(4):1841-75.

E43. Maslov LN, Popov SV, Naryzhnaya NV, Mukhomedzyanov AV, Kurbatov BK, Derkachev IA, et al. The regulation of necroptosis and perspectives for the development of new drugs preventing ischemic/reperfusion of cardiac injury. Apoptosis 2022;27(9-10):697-719.

E44. Naryzhnaya NV, Maslov LN, Popov SV, Mukhomezyanov AV, Ryabov VV, Kurbatov BK, et al. Pyroptosis is a drug target for prevention of adverse cardiac remodeling:

The crosstalk between pyroptosis, apoptosis, and autophagy. J Biomed Res. 2022;36(6):375-389.

E45. Popov SV, Maslov LN, Naryzhnaya NV, Mukhomezyanov AV, Krylatov AV, Tsibulnikov SY, et al. The role of pyroptosis in ischemic and reperfusion injury of the heart. J Cardiovasc Pharmacol Ther 2021;26(6):562-574.
